# Migration of Human Renal Tubular Epithelial Cells in Response to Physiological Electric Signals

**DOI:** 10.3389/fcell.2021.724012

**Published:** 2021-09-14

**Authors:** Linbo Guan, Ping Fan, Xinghui Liu, Rui Liu, Yu Liu, Huai Bai

**Affiliations:** ^1^Laboratory of Genetic Disease and Perinatal Medicine, Key Laboratory of Birth Defects and Related Diseases of Women and Children of the Ministry of Education, West China Second University Hospital, Sichuan University, Chengdu, China; ^2^Department of Obstetrics and Gynecology, West China Second University Hospital, Sichuan University, Chengdu, China; ^3^Division of Peptides Related with Human Disease, West China Hospital, Sichuan University, Chengdu, China; ^4^Department of Biochemistry and Molecular Biology, West China School of Preclinical and Forensic Medicine, Sichuan University, Chengdu, China

**Keywords:** renal epithelial cells, cell migration, electric fields, galvanotaxis/electrotaxis, wound healing, signal transduction

## Abstract

Restoration of proximal tubular cell integrity and function after ischemic injury involves cell migration and proliferation. Endogenous fields are present during embryonic development and wound healing. Electric field (EF)-induced effects on cell migration have been observed in many cell types. This study investigated the effect of physiological direct current EF (dc EF) on the motility of renal epithelial cells. Human renal tubular epithelial (HK-2) and human-derived renal epithelial (HEK-293) cells were exposed to dc EF at physiological magnitude. Cell images were recorded and analyzed using an image analyzer. Cell lysates were used to detect protein expression by western blot. Scratch wounds were created in monolayers of HK-2 cells, and wound areas of cells were measured in response to EF exposure. Cells migrated significantly faster in the presence of an EF and toward the cathode. Application of an EF led to activation of the Erk1/2, p38 MAPK, and Akt signaling pathways. Pharmacological inhibition of Erk1/2, p38 MAPK, and Akt impaired EF-induced migratory responses, such as motility rate and directedness. In addition, exposure of the monolayers to EF enhanced EF-induced HK-2 wound healing. Our results suggest that EFs augment the rate of single renal epithelium migration and induce cell cathodal migration through activation of Erk1/2, p38 MAPK, and Akt signaling. Moreover, exposure of the renal epithelium to EF facilitated closure of *in vitro* small wounds by enhancing cell migration.

## Introduction

Cell migration, including epithelial cell migration, is not only a key component of normal tissue homeostasis but is also crucial for wound healing and tissue regeneration (Aman and Piotrowski, 2010; Calve and Simon, 2012). Effective directional migration of endogenous renal tubular cells to the lesion is important in the wound healing process during ischemic acute kidney injury (AKI) (Nony and Schnellmann, 2003; Palmyre et al., 2014). It has been suggested that a migratory response is triggered in uninjured or sublethally injured cells to cover the exposed area of the basement membrane after cell death, followed by a proliferative response in these migrated cells to repair the lesion (Nony and Schnellmann, 2003; Palmyre et al., 2014).

The physiological electric field (EF) occurs during embryonic development (Robinson and Messerli, 1996) and wound healing (Chiang et al., 1992; Sta Iglesia and Vanable, 1998). *In vitro* studies have shown that applied small EFs regulate many crucial cellular behaviors, such as cell proliferation, cell migration, and cell differentiation. Substantial evidence has shown the potential of EFs for directing and enhancing the regrowth of damaged epithelial wounds (McCaig et al., 2009). Studies have also shown that the migration of a variety of cells, such as neural crest cells (Nuccitelli and Erickson, 1983; Stump and Robinson, 1983), fibroblasts (Erickson and Nuccitelli, 1984), neurons (Jaffe and Poo, 1979; Hinkle et al., 1981), endothelial cells (Bai et al., 2004; Zhao et al., 2004), and corneal epithelial cells (Soong et al., 1990), originating from different organs can be guided by an applied EF. These results indicate the possibility that EFs may direct renal epithelial cells to migrate *in vivo* and have implications for use in acute kidney lesions to initiate the tubular regeneration process. Therefore, it is critical to elucidate the potential mechanisms underlying this behavior.

In epithelial tissues, EF is known to increase the migration of skin epithelial cells (Yang et al., 2013), corneal epithelial cells (Farboud et al., 2000), and small intestine epithelial cells (Pu et al., 2015). However, the effects of EF on renal epithelial tubular cells have not been explored. The present study examined the effects of EF on renal epithelial cell migration since EF might be an important element for renal repair. We also determined the signaling pathways as PI3K/Akt and MAPKs (Supplementary Figure 1) that mediate this migration effect. Our results indicate that EF augments the rate of single renal epithelium migration and induces cell cathodal migration through activation of Erk 1/2, p38 MAPK, and Akt signaling. Moreover, exposure of the renal epithelium to EF facilitated closure of small wounds *in vitro* through enhanced cell migration.

## Materials and Methods

### Cell Cultures and Reagents

The human renal proximal tubular epithelial (HK-2) and human-derived renal epithelial (HEK-293) cells from the Procell Life Science and Technology (catalog nos. CL-0109 and CL-0005) were used. HK-2 and HEK-293 cells were maintained in respective DMEM/low glucose and DMEM/high glucose supplemented with 10% fetal bovine serum (FBS), 2 mM L-glutamine, penicillin (50 units/mL), and streptomycin (50 μg/mL) at 37°C in 5% CO_2_. Primary antibodies against Erk1/2, p38 MAPK, Akt (panspecific antibody and active form), and GAPDH were purchased from Cell Signaling Technology Inc. The DyeLight 680-labeled secondary antibody to rabbit IgG (H + L) was a product of LKP, Inc. BCA Protein Assay Kits were purchased from Thermo Inc. The whole protein extraction kit was from KeyGEN BioTECH.

### Electrical Stimulation

The HK-2 and HEK-293 cells were cultured in respective DMEM medium described above supplemented with 10% fetal bovine serum. The experimental setup and EF exposure protocols were similar to those reported previously (Zhao et al., 1996; Supplementary Figure 2). In brief, renal epithelial cells at a density of ∼20 × 10^4^ cells/mL for morphological analysis or 1 × 10^5^ cells/mL for protein analysis were seeded into specially made troughs formed by two parallel (2 cm apart) strips of glass coverslips (No. 1, length of 22 or 50 mm) fixed to the base of the dish with silicone grease (Dow Corning, DC4). Cells were incubated for 24–48 h (37°C, 5% CO_2_), allowing them to settle and adhere to the base of the dish, before a roof of a No. 1 coverslip was applied and sealed with silicone grease. The final dimensions of the chamber through which current was passed were 22 × 10 × 0.2 mm or 50 × 10 × 0.2 mm. Agar-salt bridges not less than 15 cm long were used to connect silver/silver-chloride electrodes in beakers of Steinberg’s solution (58 mM NaCl, 0.67 mM KCl, 0.44 mM Ca (NO_3_)_2_, 1.3 mM MgSO_4_, 4.6 mM Trizma base, pH 7.8–8.0) to pools of excess culture medium on either side of the chamber. This prevented diffusion of the electrode products into the culture medium. EFs in the physiological range of 100 and 250 mV/mm were used. Field strengths were measured directly at the beginning of, the end of and during each experiment. No fluctuations in field strength were observed. Time-lapse imaging was used to record cell migration using a time-lapse microscope (Nikon Ti-E). Cell migration was recorded for 6 h by capturing images every 5 min during the recording period.

### Quantification of Cell Behavior

Time-lapse images were analyzed using Image-Pro Plus software. Cell migration was quantified using a previously reported method (Zhao et al., 1996; Yao et al., 2009). Cell migration velocity was calculated from the full distance of cell migration at a given time. To quantify cell behavior, cells from three independent experiments were analyzed.

### Wounding and Wound-Healing Assay

The setup of the wound healing experiment was generally similar to that reported by Wang et al. (2003). Wounds were made by scratching a confluent monolayer with the pipette tip under a dissecting microscope. Twelve wounds were created on a 10 × 22-mm monolayer in each chamber, with a mean width of 600 ± 45 μm and length of 6 mm for each wound. Wound scratches were perpendicular to the long axis of the culture chamber. Time-lapse image recording was used to measure wound closure. A wound was divided by the wounding scratch into two halves. Therefore, a wound exposed to an EF perpendicular to the wound edges has both an anode-facing edge and a cathode-facing edge. The average distance of the wound edge of each half was measured using Image-Pro Plus software at various times after wound initiation. The wound edge of each half was delineated by the wound scratch and the wound edge.

### Western Blot Analysis

HK-2 cells were stimulated using EF at different time points: 5, 15, 30, and 60 min. After each time point, the medium was aspirated, and cells were lysed in 100 μl of lysis buffer (20 mM Tris-HCl, 10% glycerol, 0.2 mM EDTA, 0.137 M NaCl, 1% NP-40) supplemented with a complete protease and phosphatase inhibitor cocktail (Roche). The cellular extract was incubated for 30 min on ice and then subjected to centrifugation at 12,000 g for 15 min at 4°C. The supernatant was collected, and the amount of protein in each sample was quantitated using a BCA colorimetric assay with bovine serum albumin (BSA) as a standard. Samples containing 50 μg of total protein were loaded onto 10% SDS-polyacrylamide gels, and the electrophoresed samples were transferred onto polyvinylidene fluoride membranes as previously described (Zhang et al., 2014). Individual blots were incubated at 4°C overnight with rabbit polyclonal antibodies against Akt (9272; 1:1,000; Cell Signaling Technology, Danvers, MA), pAKT (4063; 1:1,000; Cell Signaling Technology), p38 (9212; 1:1,000; Cell Signaling Technology), pp38 (9211; 1:1,000; Cell Signaling Technology), ERK (9102; 1:1,000; Cell Signaling Technology), and pERK (4377; 1:1,000; Cell Signaling Technology), followed by incubation with a 1:10,000 dilution of fluorescently labeled goat anti-rabbit IgG antibody (072-06-15-16; KPL, Gaithersburg, MD). The intensity of bands on the western blots was quantified using Quantity One software (Bio-Rad).

### Statistical Analysis

Results are expressed as the means ± standard deviation. Values from groups were compared using the paired *t-*test or the Duncan test. A *P-*value < 0.05 was considered statistically significant. At least three independent experiments were performed for each condition unless stated otherwise.

## Results

### Small EFs Direct the Migration of Renal Epithelial Cells Toward the Cathode

**FIGURE 1:**
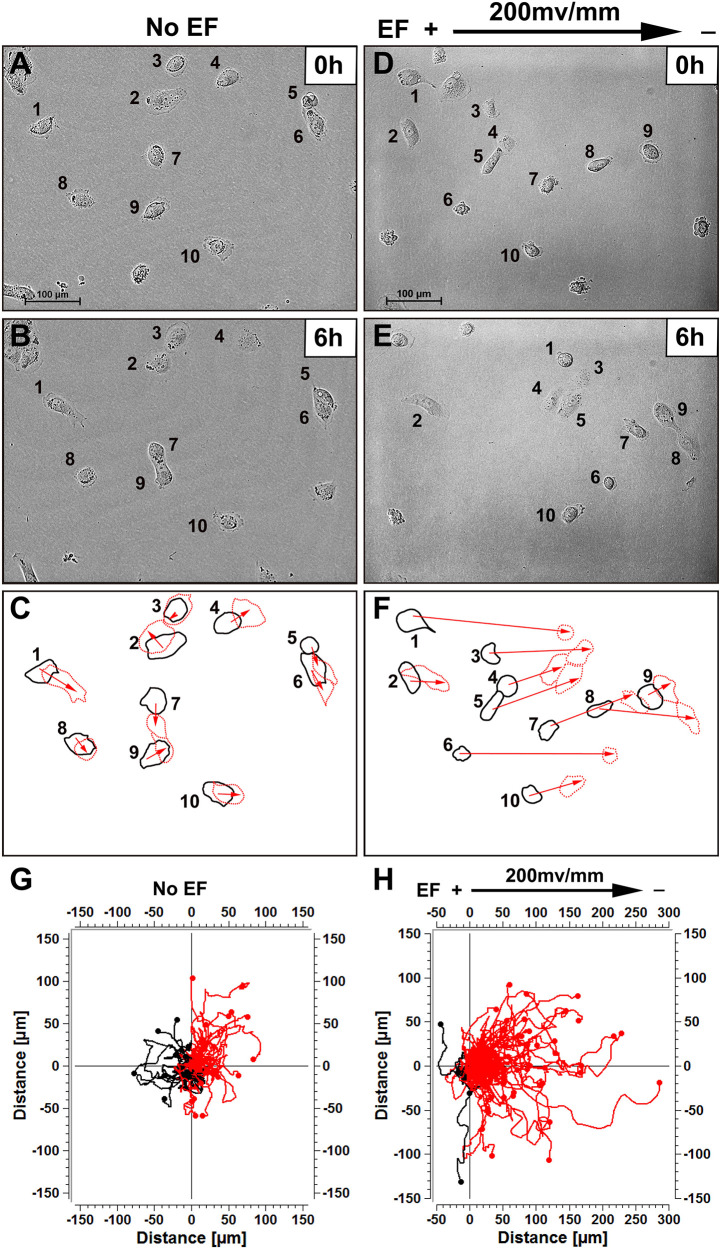
Human renal tubular epithelial cells migrate cathodally in small physiological electric fields. **(A,B)** Random migration of cells over a 6 h period without EF stimulation. **(D,E)** Cells migrated to the cathode right in response to EF stimulation over 6 h (see “Supplementary Video 1”). Cells are identified by numbering. **(C,F)** Show the outlines of the cells at the beginning and end of each experiment, with migration direction indicated by the arrows. In **(G,H)** each frame shows the superimposed migration tracks of 60–125 HK-2 cells from three different experiments. The position of all cells at *t* = 0 min is represented by the origin (0, 0). Each line represents the migration track of one single cell over a 6-h period. Scale bar = 100 μm.

The effect of an EF on directionality in HK-2 and HEK-293 cells was determined. When cultured without EF, renal epithelial cells migrated in random directions. When cultured in a physiological EF (200 mV/mm), the cells showed evident directional migration. HK-2 cells migrated toward the cathode (Figure 1 and Supplementary Video 1). In addition, HEK-293 cells also showed similar responsive feature in the EF culture condition and moved toward the cathode direction (Supplementary Figure 3 and Supplementary Video 2). Cell migration was quantified as previously described (Zhao et al., 1996; Yao et al., 2009).

### Directional Migration of Renal Epithelial Cells Cultured in EFs Is Voltage Dependent

**FIGURE 2:**
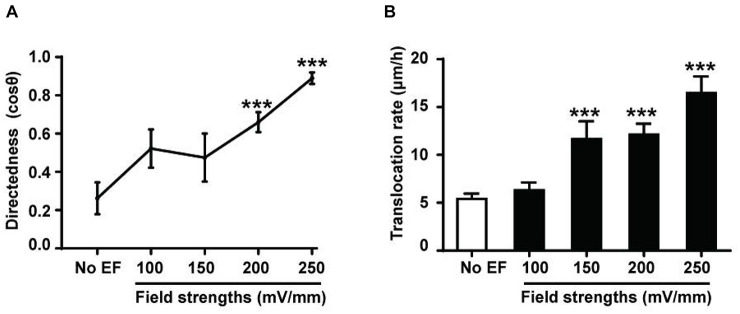
Voltage dependence of EF-directed migration and enhanced migration rate of human renal tubular epithelial cells. Directedness and rate of cell migration of HK-2 cells cultured in EFs (see section “Materials and Methods”) were calculated during a 6-h period. Cathodal migration of the cells was voltage dependent **(A)**, and an increase in the migration rate was also voltage dependent **(B)**. The cell numbers included in the analysis at individual field strengths of 0–250 mV/mm are 28–81. ***P < 0.001, compared to the control with no EF (0 mV). Values are shown as means ± SEM.

EF-directed cell migration was dependent on voltage. In the EF group, after a 6 h exposure to an EF stimulation of 100, 150, 200, or 250 mV/mm, the directedness was 0.52 ± 0.10, 0.47 ± 0.13, 0.66 ± 0.05, and 0.89 ± 0.03, respectively (Figure 2A). The cell migration velocities of HK-2 cells in the absence (No EF) and presence of EFs were quantified and compared (Figure 2B). The translocation rate of cells without EF stimulation was 5.41 ± 0.54 μm/h, which was increased to 11.64 ± 1.86 μm/h after the cells were subjected to an EF of 150 mV/mm for 6 h (*p* < 0.001). The translocation rate also changed significantly after the cells were subjected to EFs of 200 mV/mm (12.12 ± 1.14 μm/h) and 250 mV/mm (16.46 ± 1.75 μm/h) for 6 h (both *p* < 0.001). In addition, the cell migration velocity of HEK-293 cells in the EF culture was also increased as compared to the no EF treated control cells (Supplementary Figure 3 and Supplementary Video 2) (*p* < 0.001).

### Effects of EFs on Migration of Cells in a Wounded Monolayer

**FIGURE 3:**
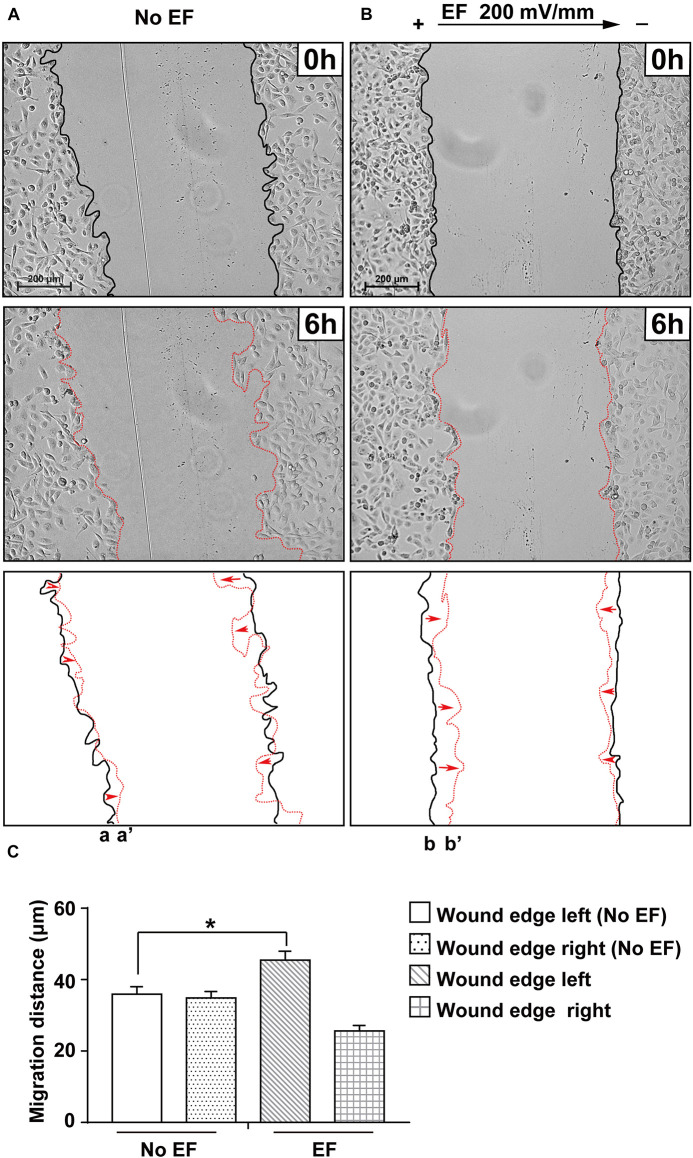
Effect of EF stimulation on wounded human renal tubular epithelial monolayer migration. Confluent renal cellular monolayers were wounded and cultured in EF for 6 h at a field strength of 200 mV/mm. The presence of EF significantly enhanced the cell sheet of the cathode-facing side moving toward the cathode (b to b’) during a 6 h culture period **(B)**, while the no EF control showed a similar wound closure rate of cell sheets on either side (toward the center of the culture) **(A)**. Polarity is as indicated; the cathode is at right. Bottom panels show the outlines of the wounded monolayers front at the beginning and end of each experiment with migration direction indicated by the arrows. Scale bar = 200 μm. *P < 0.05, compared to the same edge of the control with no EF (0 mV). **(C)** Quantification of the cell migration distance of wound edges in different treatment groups (with and without EF stimulation).

To examine the role of EFs in regulating renal epithelial cell restitution, a single wound was created in a confluent renal epithelial HK-2 cell monolayer using a pipette tip. Cell monolayers were cultured with EF (200 mV/mm) and with no EF. As shown in Figure 3, EF significantly increased the rate of wound closure (also see Supplementary Video 3). The migration distance of renal epithelial monolayers at left edges was measured as 35.88 ± 2.08 and 45.46 ± 2.53 μm in cells cultured with no EF (0 mV/mm) and under EFs at 6 h, respectively (*p* < 0.01), while the right edges showed no significant difference in migration distance between the two groups (0 mV/mm vs. 200 mV/mm: 35.87 ± 2.31 and 32.73 ± 1.55 μm, *P* > 0.05). Therefore, EF exposure significantly enhances renal epithelial cell restitution, especially on the cathode-facing side (left) of the wounded monolayer. In contrast, the migration distance of a cell monolayer for 6 h on the left edge without exposure to EFs was much shorter.

### Effects of EFs on the Activation of Erk1/2, p38 MAPK, and Akt

**FIGURE 4:**
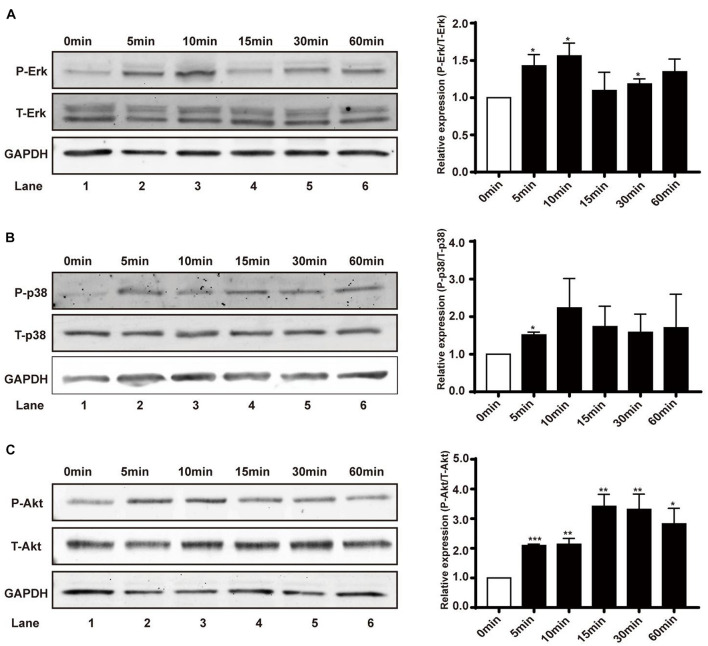
Effect of EF on activation of Erk1/2, p38 MAPK, and Akt signaling pathways in human renal tubular epithelial cells. **(A–C)** Protein expression of Erk1/2 and Erk1/2-phosphorylation, p38 and p38-phosphorylation, and Akt and Akt-phosphorylation in different treatment groups (before or after EF stimulation) were detected by western blotting with anti-Erk1/2 and anti-p-Erk1/2, and anti-p38 and anti-p-p38, and anti-Akt and anti-p-Akt. GAPDH was used as an internal control. Data are presented as the mean ± SEM of at least three biological replicates, *p < 0.05, **P < 0.01, or ***P < 0.001, compared with control before EF treatment (0 min).

Erk1/2, p38 MAPK, and Akt have been implicated as signaling molecules involved in the early response of cells to multiple stimuli. In this study, renal epithelial cells were treated with an EF (200 mV/mm) for varying time periods (0, 5, 10, 15, 30, and 60 min), and cell lysates collected at specific time points were subjected to western blotting. There was obvious early activation of Erk1/2, p38 MAPK, and Akt at the 5 min time-point, as measured by levels of phosphorylation in renal epithelial cells (Figures 4A–C), suggesting primary activation of these signaling pathways in response to EF stimulation. JNK, another member in the family of MAPKs, showed no activation after 1 h under EF exposure (data not shown).

### Erk1/2, p38 MAPK, and Akt Signaling Are Involved in the Migration Response

**FIGURE 5:**
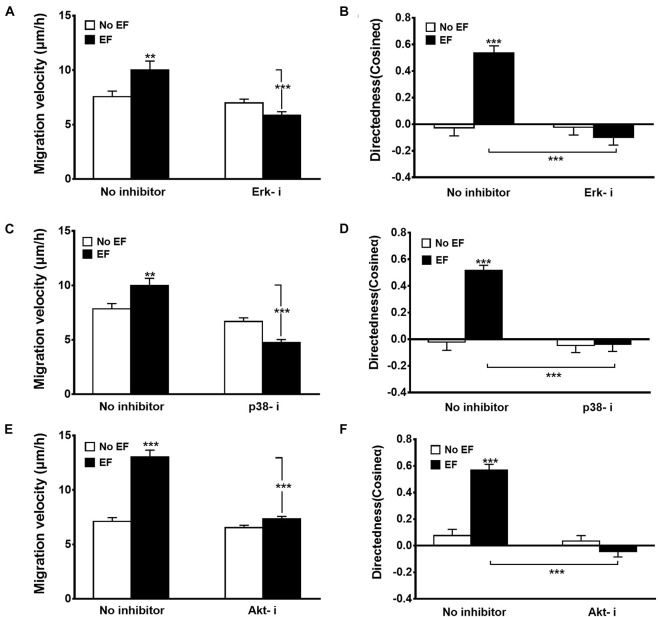
Effects of various inhibitors on EF-induced cellular migration. Inhibition of Erk1/2 (Erk1/2-i), p38 (p38-i), and Akt (Akt-i) significantly decreased migration velocity **(A,C,E)** and directedness **(B,D,F)** responses. Erk1/2-i, Erk1/2 MAPK inhibitor U0126 (20 μM); p38-i, p38 inhibitor SB203580 (50 μM); Akt-i, Akt inhibitor (10 μM). HK-2 cells were subjected to EFs of 200 mV/mm for 6 h. *n* = 64–328 from at least three independent experiments. **P < 0.01, ***P < 0.001, significantly different from values as indicated.

To determine the significance of Erk1/2, p38 MAPK, and Akt activation in EF-mediated increases in migratory capacity (migration velocity, Figures 5A,C,E, and directedness, Figures 5B,D,F) of renal epithelial cells, specific Erk inhibitor (Erk-i, U0126), p38 inhibitor (p38-i, SB203580), and Akt inhibitor (Akt-i, MK-2206 2HCL) were used. None of the inhibitors displayed toxic effects in renal epithelial cells nor retarded cell growth (data not shown). In response to EF treatment at 200 mV/mm, there were obvious responses of the Erk- i-, p38-i- and Akt-i-untreated cells (Figure 5). However, cells treated with Erk1/2, p38, and Akt inhibitors exhibited significantly inhibited cellular responses (percent inhibition of migration speed: 47.7, 58.5, and 56.3%, respectively), including cell migration direction and speed (Figure 5) (all *p* < 0.001). There were no significant differences of cellular responses between Erk1/2, p38, and Akt inhibitor treated control cells and respective no treated control cells, indicating cellular responses were not impaired in the presence of these inhibitors under control condition. Additional study also showed the role of PI3K in EF-stimulated migration response of the renal epithelial cells with the PI3K inhibitor LY294002 (Supplementary Figure 4). Moreover, pharmacological inhibitors of Erk, p38, and Akt blocked the scratch assay enhanced wound response to the EF (Supplementary Figure 5).

## Discussion

In the present study, we provide evidence that EF markedly induces the migration of human renal epithelial cells, which extends our knowledge concerning EF-responsive cell types (McCaig and Zhao, 1997). We demonstrated that renal epithelial cells migrated significantly faster in the presence of an EF and toward the cathode. Application of an EF led to activation of the Erk1/2, p38 MAPK, and Akt signaling pathways. Inhibition of Erk1/2, p38, and Akt activities reduced the ability of EFs to enhance the rate of HK-2 migration and inhibited EF-induced cathodal directionality. In addition, exposure of the monolayers to EF enhanced EF-induced epithelial cell wound healing.

Small DC EFs stimulate the motility of many cell types in humans and other mammalian species (McCaig and Zhao, 1997). Studies have demonstrated that small DC EFs are an overriding guidance cue that directs corneal epithelial cell migration in wound healing. In endothelial cells, small DC EFs induce cell migration, cell alignment, cell elongation and production of VEGF and IL-8 (Bai et al., 2004; Zhao et al., 2004), potentially providing a new approach to modulate angiogenesis. Our findings demonstrated for the first time that EFs enhance HK-2 cell migration/motility and induce cell directionality, in addition to the significant effect on HEK-293 cells, implicating EFs in renal epithelial cell function. Tentatively, we propose that *in vivo* renal epithelial cell migration could be significantly enhanced by EF stimulation, and therefore EF has therapeutic potential for abnormal renal cell migration/motility related to some complications of renal organ injury (Nony and Schnellmann, 2003; Palmyre et al., 2014). This observation indicates that EFs might represent a novel cue involved in mediating the migration of renal epithelial cells.

Many studies have shown that the mechanism of transduction of electrical signals to cells likely occurs via activation of cell signaling pathways (Sato et al., 2009). PI3-kinase/Akt are involved in cell migration (Vanhaesebroeck et al., 2012), and EF stimulates activation of PI3k/Akt during EF-induced cellular migratory behaviors in various types of cells (Bai et al., 2004; Zhao et al., 2004; Meng et al., 2011; Li et al., 2013; Jeon et al., 2019). In dictyostelium cells PI3K, together with the catalytic domains of sGC and GbpC are involved in cathodal migration (Sato et al., 2009), whereas treatment with PI3K inhibitor LY294002 reduces the cathodal migration in EF (Arocena et al., 2010). Meng et al. (2011) reported that in embryonic and adult neural progeniter cells physiological EFs can trigger the redistribution of PIP3, the down-stream effectors of PI3K, and the colocalization with actin at the leading edge of the electrotacxing cells in the presence of growth factors. A study with *ex vivo* model demonstrated that the involvement of PI3K signaling pathway in electrotaxis is also evidenced. In organotypic spinal cord slice culture the grafted neural progenitor cells (NPCs) migrate directionally toward the cathode of EF, along with the increased phosphorylation of PI3K downstream effector Akt and PIP3 expression level. In the present investigation, EFs stimulated phosphorylation of Akt, the downstream target of PI3-kinase. Furthermore, EF-mediated cell migration was prevented by administration of an Akt inhibitor. The PI3-kinase/Akt pathway regulates actin cytoskeleton reorganization, possibly involving activation of small Rho guanosine triphosphatases (GTPases) that produce protrusions of the plasma membrane and formation of lamellipodia (Liu et al., 2019). Increasing evidence suggests that growth factor-induced modulation of the actin cytoskeleton is essential for cell migration. One possible mechanism by which EF may facilitate renal epithelial cell migration is through activation of the PI3-kinase/Akt pathway and subsequent modulation of the actin cytoskeleton.

The ERK family of MAP kinases also plays an important role in cell migration. One of the downstream effectors of activated ERK is myosin light chain kinase (MLCK). Through activation of MLCK, ERK is thought to stimulate cell migration (Klemke et al., 1997). ERK is a common downstream signaling protein activated after growth factor receptor stimulation, and ERK can be activated downstream of PI3-kinase activation. Studies with fibrosarcoma and glioma cells revealed that EF triggers generation of hydrogen preoxide and superoxide through the activation of nicotinamide adenine dinucleotide phosphate (NADPH) oxidase. The overly superoxide produced during this process facilitates the phosphorylation of ERKs, which results in the MAPK/ERK activation, cytoskeleton reorganization, and directional migration (Li et al., 2012, 2013; Wolf-Goldberg et al., 2013). Several other reports also showed the involvement of ERKs pathway in electrotaxis, together with other cellular responses to EF stimulation (McBain et al., 2003; Chernyavsky et al., 2005; Hart et al., 2013). Exposure of human renal epithelial cells to EF led to phosphorylation of ERK 1/2, and exposure to U0126 significantly reduced the ability of EF to augment cell migration and inhibited EF-induced cathodal directionality, suggesting the involvement of ERK activation in the actions of EF.

p38 MAPK can be activated by MAPK kinases (MKKs) upon exposure to different stimuli, such as environmental stress, inflammatory cytokines and growth factors (Zarubin and Han, 2005; Cuenda and Rousseau, 2007). The role of p38 in cell migration was first observed in endothelial cells stimulated by vascular endothelial growth factor (VEGF), with the underlying mechanism showing that p38 regulated cell movement by inducing cytoskeletal rearrangement through the activation of HSP27, the p38 substrate (Rousseau et al., 1997). A recent study demonstrated that p38 MAPK promotes the migration and metastatic activity of BRAF-mutated melanoma cells by inducing the degradation of PMCA4b, one of the key regulators involved in the maintenance of intracellular Ca^2+^ concentrations and linked to the modulation of cytoskeletal rearrangement (Naffa et al., 2020). In the present study, we showed that EF enhanced phosphorylation of p38, and inhibition of p38 MAPK led to a significant inhibition of EF-mediated migration speed and directionality of renal cell migration. These results suggest that the p38 MAPK pathway is also implicated in EF-mediated renal cell functions, such as cell motility. How p38 activation response to EF stimulation is controversial. One study showed that p38 pathway and its downstream transcription factors, AP-1, were negatively modulated by EF in lipopolysaccharide (LPS)-induced inflammatory response (Jeong et al., 2013), whereas Zhao et al reported that EF triggered clear phosphorylation in keratinocytes and neutrophils (Zhao et al., 2006). These conflicting results suggest that potential role of MAPKs super family in EF stimulation could be cell type dependent which requires further in-depth exploration.

Of note, there is evidence that the p38 MAPK pathway can be activated in response to different types of EFs. For instance, it has been reported that DC EF activates several intracellular pathways, including p38 MAPK, in embryonic stem cells and induces endothelial differentiation (Sauer et al., 2005). Another study reported that 900 MHz mobile phone radiation activated the heat shock protein 27 (Hsp27)/p38 MAPK pathway in human endothelial cells (Leszczynski et al., 2002). Additionally, different types of electromagnetic fields have been shown to affect the activation of p38 MAPK, as well as other intracellular pathways in several other cell types (Nie and Henderson, 2003; Zhao et al., 2006; Friedman et al., 2007).

All cell types and intracellular organelles maintain transmembrane electrical potentials owing to asymmetric ion transport. For instance, in mammalian corneal epithelium, naturally occurring EFs in tissue arise from polarized ion transport, i.e., enriched Na^+^ channels (and Cl^–^ transporters) in the apical domain of the cell (McCaig et al., 2009). In human adipose tissue-derived stem cells, there are channels for a Ca^2+^-activated K^+^ current, a transient outward K^+^ current, a delayed rectifier-like K^+^ current, and a tetrodotoxin-sensitive transient inward Na^+^ current (Bai et al., 2007). It has been suggested that in almost all systems, crucial cellular behaviors, such as division, migration and differentiation, takes place within an extracellular microenvironment in which standing voltage gradients persist for several hours or even for a few days (McCaig et al., 2005; Levin, 2007). Based on the above reports and the present finding that renal epithelial cells are responsive to EF signaling, we speculate that EF might exert a profound influence on renal epithelial cell function via direct activation of ion channels. An interesting area for future work might be to investigate whether ion channels, including the epithelial Na^+^ channel on renal epithelial cells, are involved in the transduction of electrical stimuli and thus have potential significance in EF-mediated cellular functions linked to critical renal organ functions.

Evidence has shown enhanced wound healing in response to EF application to skin wounds (Gentzkow, 1993). A previous study reported that applied DC EF influenced the healing of lens epithelial cell monolayer wounds and that this effect varied with the polarity of the EF (Wang et al., 2003). In this study, we showed a similar effect of the EF-stimulated response of renal epithelial monolayer wounds. The strips of cells created by the scratch wounds had anode- and cathode-facing wound edges. Edges facing the cathode closed faster than edges facing the anode. The latter closed eventually after enlarging (Figure 3). There is convincing evidence that some signaling mediators of DC EF-directed cell migration are involved. The relationship between the collected electric stimulation of renal epithelial cells and the activation of intracellular signaling pathways remains to be further elucidated.

In conclusion, the present study provides the first evidence that a physiological level of DC EF stimulates the migration of renal tubular epithelial cells, extending our knowledge concerning EF-responsive cell types (Ferrier et al., 1986; Zhao et al., 1996, 2004; Chao et al., 2000; Bai et al., 2004; Chen et al., 2021). We suggest that EF augments the rate of single renal epithelium migration and induces cell cathodal migration through activation of the Erk 1/2, p38 MAPK, and Akt signaling pathways. Moreover, exposure of the renal epithelium to EF facilitated closure of *in vitro* small wounds through enhanced cell migration. These findings may have potential implications for the treatment of ischemic renal diseases and for research into renal regeneration.

## Data Availability Statement

The original contributions presented in the study are included in the article/Supplementary Material, further inquiries can be directed to the corresponding author/s.

## Author Contributions

LG took part in most of the experiments and analyzed the data. PF and RL performed part of the experiments. XL and YL helped with some of the experimental design, as well as with writing and analysis of the data. HB performed part of the experiments, analyzed the results, and wrote the manuscript. All authors have read and approved the final manuscript.

## Conflict of Interest

The authors declare that the research was conducted in the absence of any commercial or financial relationships that could be construed as a potential conflict of interest.

## Publisher’s Note

All claims expressed in this article are solely those of the authors and do not necessarily represent those of their affiliated organizations, or those of the publisher, the editors and the reviewers. Any product that may be evaluated in this article, or claim that may be made by its manufacturer, is not guaranteed or endorsed by the publisher.

## Supplementary Material

Supplementary Figure 1 Schematic graph showing signaling pathways in EF stimulated cells. The EF-regulated signaling activation includes the redistribution of ion channels transport proteins (NaKA, NHE3) and receptors (AchR, NMDAR) at the cell membrane level, which triggers the cytoskeletal polarization, stimulates the activation of relevant intracellular signaling pathways (PI3K/Akt, PI3K/Rho; ERK, p38, JNK MAPKs; PTEN, sGC/GbpC, etc.).

Supplementary Figure 2 A schematic diagram showing the experimental design of the culture chamber and field application and the method of quantification of cell orientation. **(A)** Chamber constructed within a tissue culture plastic dish viewed from above. **(B)** Side-on view including DC power supply and Ag/AgCl electrodes isolated from the culture chamber using agar-gelled salt bridges. **(C)** Measurement of the electric field using an electric meter.

Supplementary Figure 3 Human renal epithelial HEK-293 cells migrate cathodally in small physiological electric fields. **(A,B)** Random migration of cells over a 6 h period without EF stimulation. **(D,E)** Cells migrated to the cathode right in response to EF stimulation over 6 h (see Supplementary Video 2). Cells are identified by numbering. **(C,F)** show the outlines of the cells at the beginning and end of each experiment, with migration direction indicated by the arrows. **(G,H)** Directedness and rate of cell migration of HEK-293 cells cultured in EFs and No EF (control) were calculated during a 6-h period. Cathodal migration of the cells and an increase in the migration rate were evident **(G,H)**. The cell numbers included in the analysis in EF stimulation and control groups are 20 and 19 from two different experiments, respectively. *^∗∗∗^P* < 0.001, compared to the control with no EF (0 mV). Values are shown as means ± SEM.

Supplementary Figure 4 Effects of PI3K inhibitor on EF-induced cellular migration. Inhibition of PI3K (PI3K-i) significantly decreased migration velocity **(A)** and directedness **(B)** responses. PI3K inhibitor LY294002 (50 μM). HK-2 cells were subjected to EFs of 200 mV/mm for 6 h (*n* = 62–66 cells). ^∗∗∗^*P* < *0.001*, significantly different from values as indicated.

Supplementary Figure 5 Effects of various inhibitors on EF-induced wound healing response of HK-2 cells. Inhibition of Erk1/2 (Erk1/2-i), p38 (p38-i), and Akt (Akt-i) significantly decreased the cell sheet of the cathode-facing side moving toward the cathode (c to c’, d to d’, and e to e’) during a 6 h culture period **(C–E)**, while the no inhibitor treated cells in the presence of EF **(B)** showed the cell sheet of the cathode-facing side moving toward the cathode (b to b’). The no EF control showed a similar wound closure rate of cell sheets on either side (toward the center of the culture) **(A)**. Polarity is as indicated; the cathode is at right. Bottom panels show the outlines of the wounded monolayers front at the beginning and end of each experiment with migration direction indicated by the arrows. Scale bar = 200 μm. ^∗^*P* < 0.05, compared to the same edge of the control with no EF (0 mV). **(F)** Quantification of the cell migration distance of wound edges (left, cathode facing side) in different treatment groups.

Supplementary Video 1 Real-time imaging of live cells showing cathodal migration and enhanced motility of human renal tubular epithelial cells in culture exposed to an EF of 200 mV/mm (over 6 h).

Supplementary Video 2 Real-time imaging of live cells showing cathodal migration and enhanced motility of human renal epithelial HEK 293 cells in culture exposed to an EF of 200 mV/mm (over 6 h).

Supplementary Video 3 Real-time imaging of live wounded monolayer cells showing a significantly increased rate of wound closure, especially on the cathode-facing side (left) of the wounded monolayer of human renal tubular epithelial cells in culture exposed to an EF of 200 mV/mm (over 6 h).
