# Supplementary figures and images for: Migration of Human Renal Tubular Epithelial Cells in Response to Physiological Electric Signals
Source: Front Cell Dev Biol. 2021 Sep 14;9:724012. doi: 10.3389/fcell.2021.724012 (PMC8476913; doi:10.3389/fcell.2021.724012)

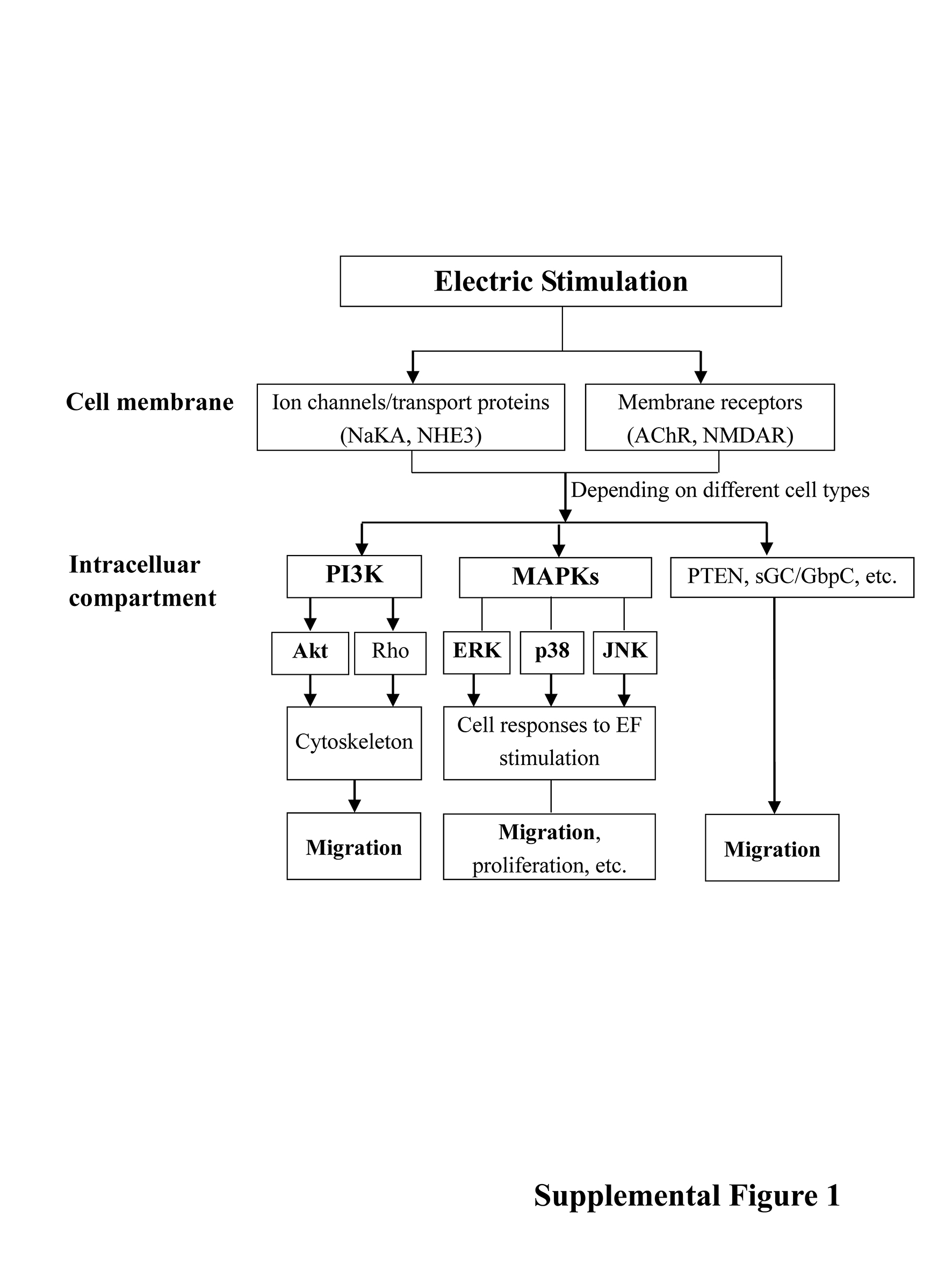

Supplement: Supplementary Figure 1 — Schematic graph showing signaling pathways in EF stimulated cells. The EF-regulated signaling activation includes the redistribution of ion channels transport proteins (NaKA, NHE3) and receptors (AchR, NMDAR) at the cell membrane level, which triggers the cytoskeletal polarization, stimulates the activation of relevant intracellular signaling pathways (PI3K/Akt, PI3K/Rho; ERK, p38, JNK MAPKs; PTEN, sGC/GbpC, etc.). [file Image_1.JPEG]

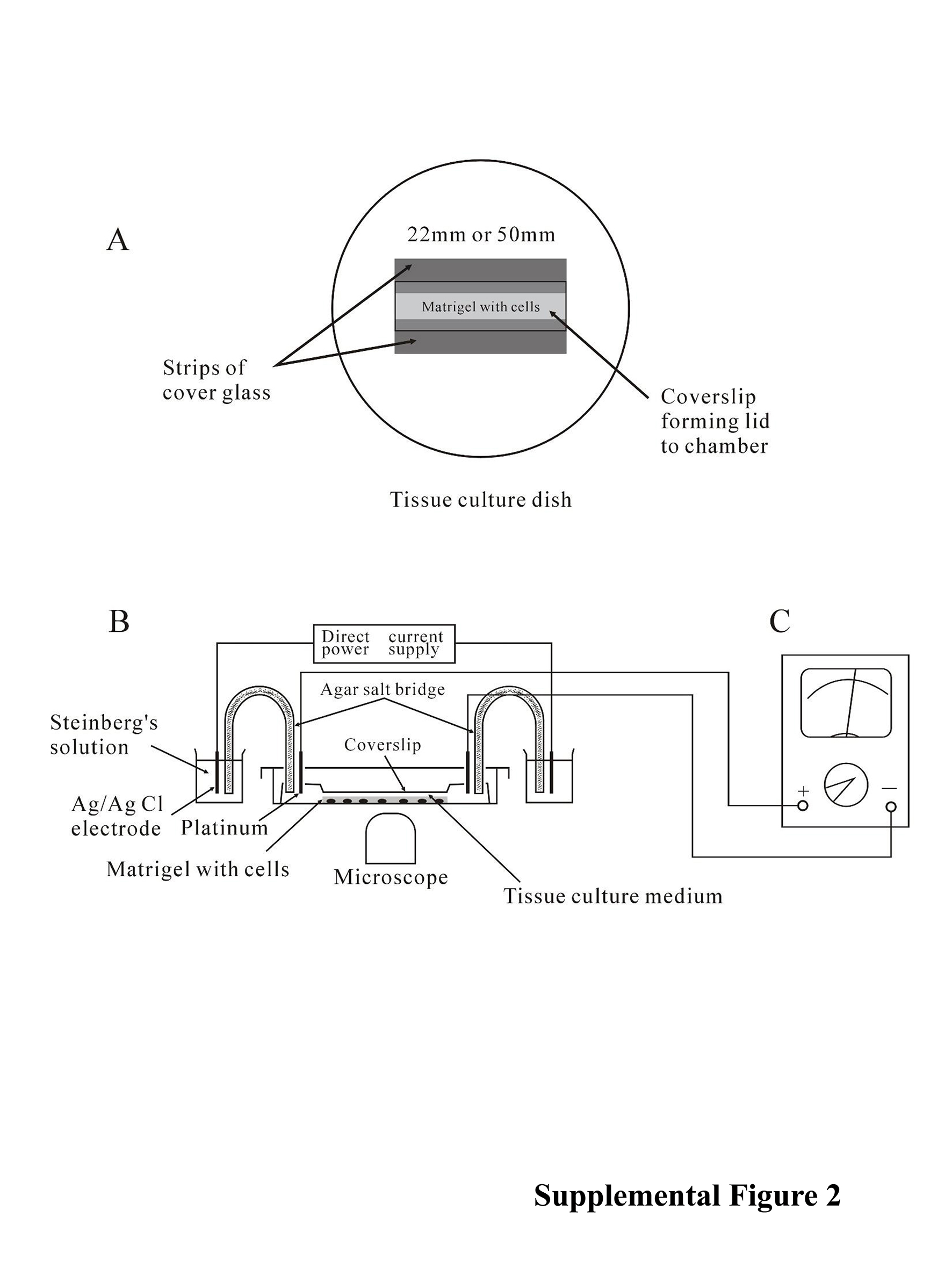

Supplement: Supplementary Figure 2 — A schematic diagram showing the experimental design of the culture chamber and field application and the method of quantification of cell orientation. (A) Chamber constructed within a tissue culture plastic dish viewed from above. (B) Side-on view including DC power supply and Ag/AgCl electrodes isolated from the culture chamber using agar-gelled salt bridges. (C) Measurement of the electric field using an electric meter. [file Image_2.JPEG]

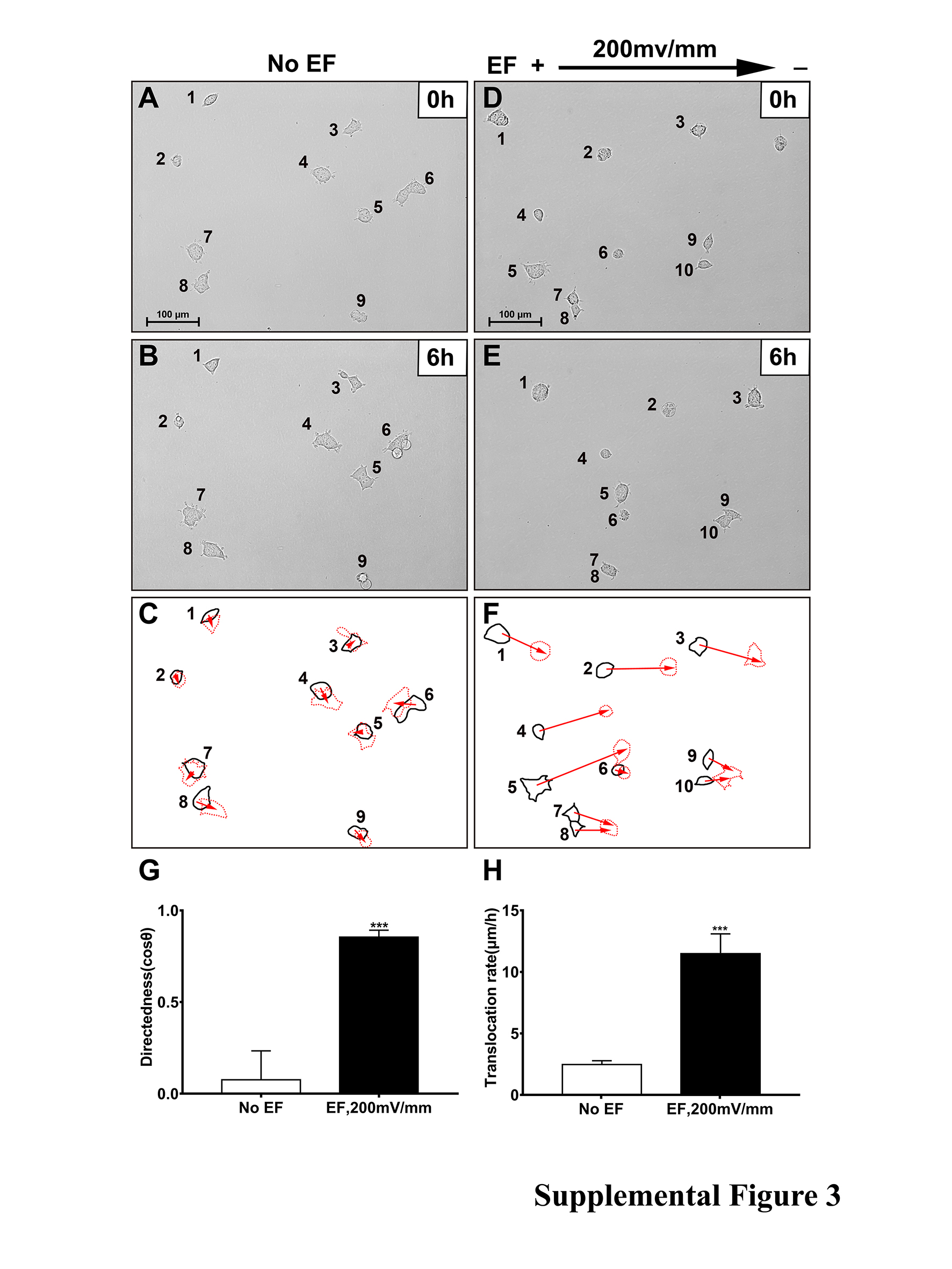

Supplement: Supplementary Figure 3 — Human renal epithelial HEK-293 cells migrate cathodally in small physiological electric fields. (A,B) Random migration of cells over a 6 h period without EF stimulation. (D,E) Cells migrated to the cathode right in response to EF stimulation over 6 h (see Supplementary Video 2). Cells are identified by numbering. (C,F) show the outlines of the cells at the beginning and end of each experiment, with migration direction indicated by the arrows. (G,H) Directedness and rate of cell migration of HEK-293 cells cultured in EFs and No EF (control) were calculated during a 6-h period. Cathodal migration of the cells and an increase in the migration rate were evident (G,H). The cell numbers included in the analysis in EF stimulation and control groups are 20 and 19 from two different experiments, respectively. ∗∗∗P < 0.001, compared to the control with no EF (0 mV). Values are shown as means ± SEM. [file Image_3.JPEG]

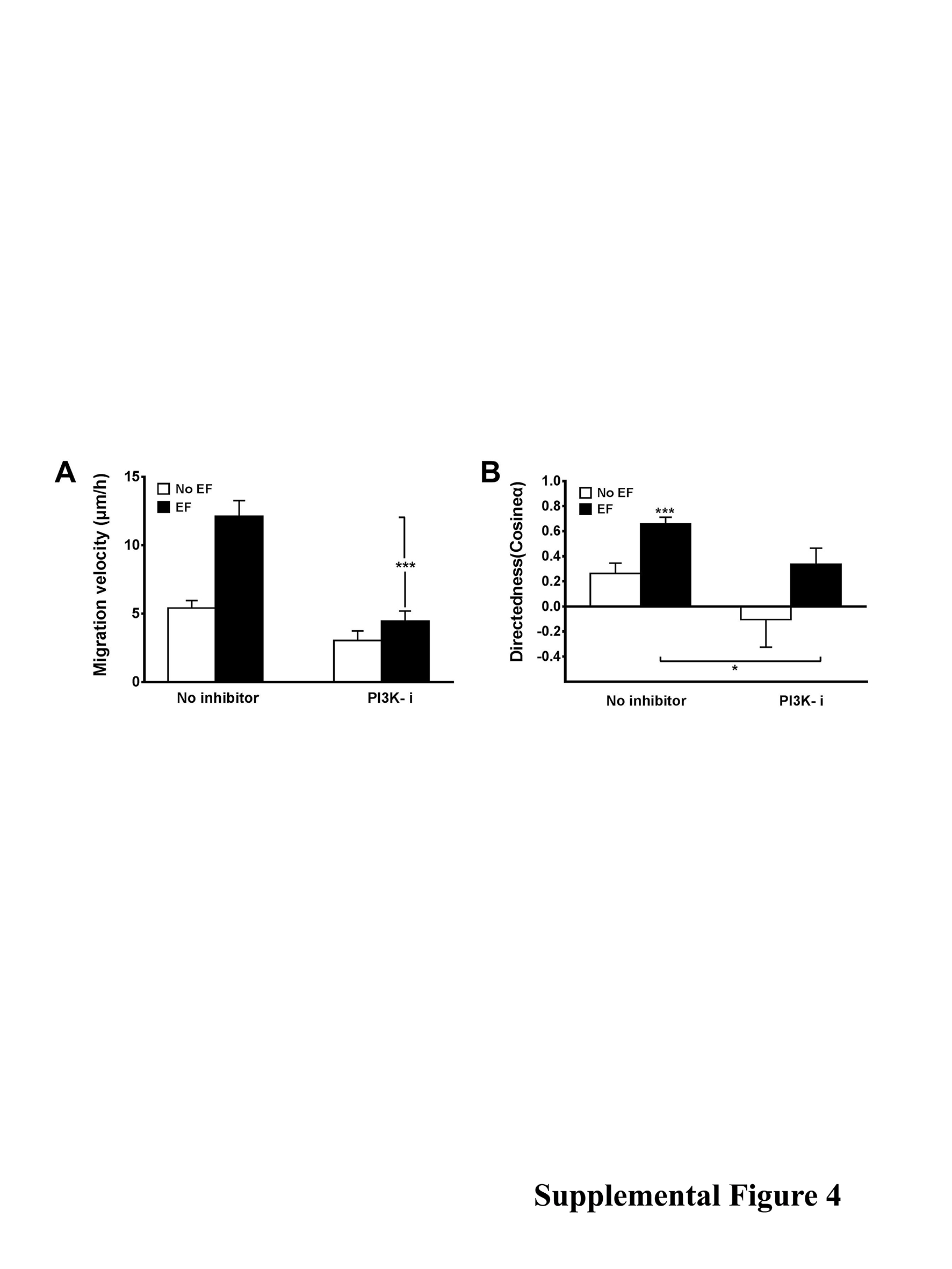

Supplement: Supplementary Figure 4 — Effects of PI3K inhibitor on EF-induced cellular migration. Inhibition of PI3K (PI3K-i) significantly decreased migration velocity (A) and directedness (B) responses. PI3K inhibitor LY294002 (50 μM). HK-2 cells were subjected to EFs of 200 mV/mm for 6 h (n = 62–66 cells). ∗∗∗P < 0.001, significantly different from values as indicated. [file Image_4.JPEG]

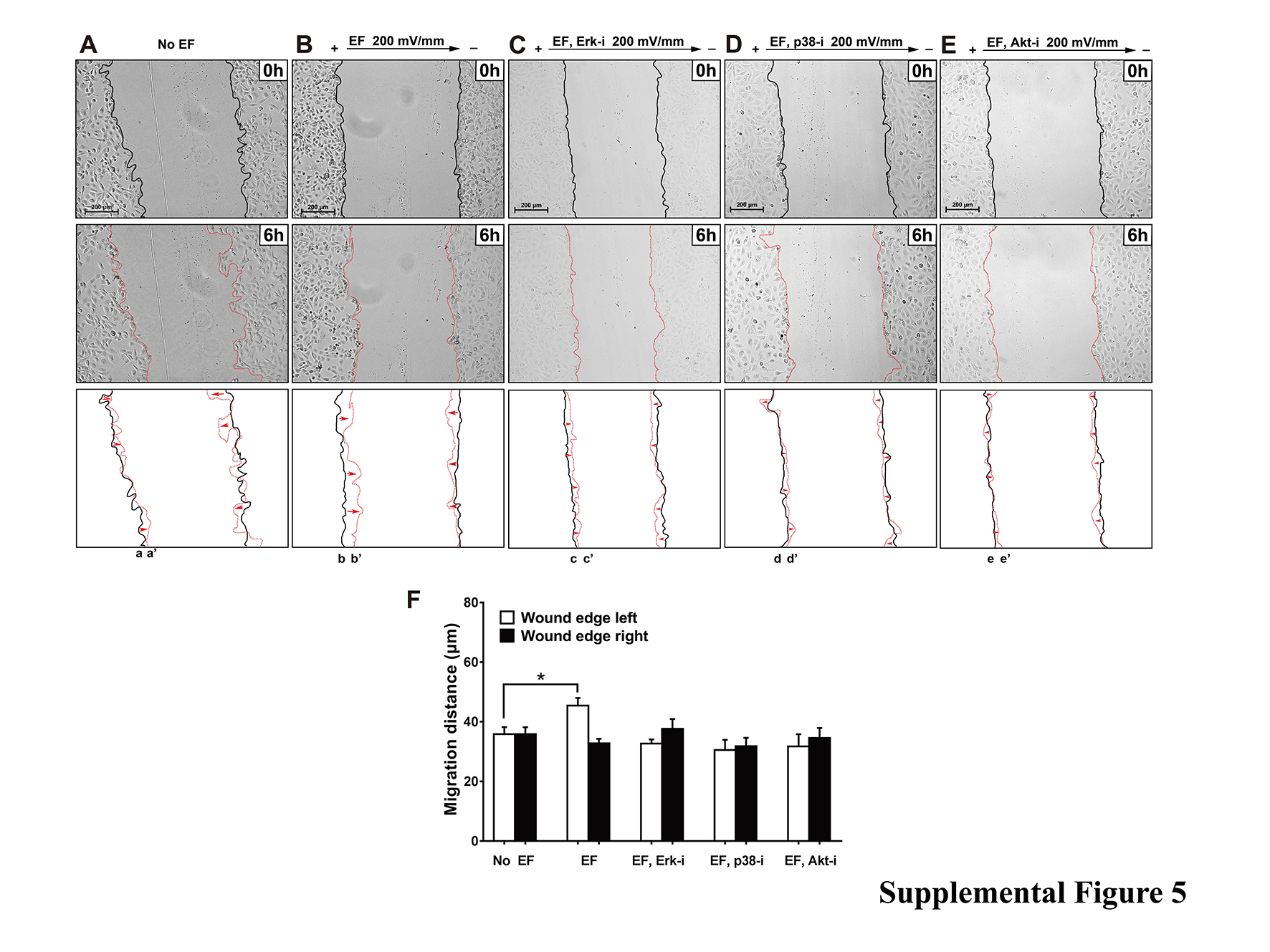

Supplement: Supplementary Figure 5 — Effects of various inhibitors on EF-induced wound healing response of HK-2 cells. Inhibition of Erk1/2 (Erk1/2-i), p38 (p38-i), and Akt (Akt-i) significantly decreased the cell sheet of the cathode-facing side moving toward the cathode (c to c’, d to d’, and e to e’) during a 6 h culture period (C–E), while the no inhibitor treated cells in the presence of EF (B) showed the cell sheet of the cathode-facing side moving toward the cathode (b to b’). The no EF control showed a similar wound closure rate of cell sheets on either side (toward the center of the culture) (A). Polarity is as indicated; the cathode is at right. Bottom panels show the outlines of the wounded monolayers front at the beginning and end of each experiment with migration direction indicated by the arrows. Scale bar = 200 μm. ∗P < 0.05, compared to the same edge of the control with no EF (0 mV). (F) Quantification of the cell migration distance of wound edges (left, cathode facing side) in different treatment groups. [file Image_5.JPEG]
